# Identification of cancer driver mutations in liquid-based cytology samples for the screening of endometrial diseases

**DOI:** 10.1038/s44276-023-00020-y

**Published:** 2023-11-02

**Authors:** Motoki Matsuura, Kiyoko Takane, Kiyoshi Yamaguchi, Tsuneo Ikenoue, Seira Hatakeyama, Shoko Kurokawa, Masato Tamate, Taishi Akimoto, Masahiro Iwasaki, Shintaro Sugita, Tadashi Hasegawa, Yasunori Ota, Tsuyoshi Saito, Yoichi Furukawa

**Affiliations:** 1 Department of Obstetrics and Gynecology, Sapporo Medical University, Sapporo, 060-8543 Japan; 2 Division of Clinical Genome Research, Advanced Clinical Research Center, The Institute of Medical Science, The University of Tokyo, Tokyo, 108-8639 Japan; 3 Department of Surgical Pathology, Sapporo Medical University, Sapporo, 060-8543 Japan; 4 Department of Diagnostic Pathology, Research Hospital, The Institute of Medical Science, The University of Tokyo, Tokyo, 108-8639 Japan

## Abstract

**Background:**

Endometrial cancer (EC) is one of the leading causes of cancer death among women and early detection is crucial for its successful treatment. We previously showed that cytological examination combined with genetic analysis using liquid-based cytology (LBC) samples improved the diagnostic sensitivity of EC.

**Methods:**

Among 218 individuals who underwent endometrial screening by LBC, 208 samples were analysed by cancer-panel sequencing. Excluding 13 samples with low sequence coverage, we further analysed 195 samples.

**Results:**

Among the 195 women analysed, 39 and 15 were eventually shown to suffer from malignant endometrial and non-endometrial neoplasms, respectively. Driver mutation(s) were found in nine of the 15 cytology-negative patients with endometrial malignancy, corroborating that combination of cytological and genetic analyses should increase the sensitivity of the diagnosis of malignant endometrial neoplasms. Importantly, driver alterations were found in 32 of 125 women without malignant or premalignant diseases, which raises caution for the interpretation of genetic alterations detected in the endometrial samples. Comparison between the driver mutations in the 32 subjects and those in the 29 endometrial malignancies unveiled that the number of mutations and mutations in *PTEN*, *CTNNB1*, and *TP53* may be applied for the assessment and prediction of development of EC.

**Conclusions:**

The genetic analysis combined with liquid-based cytology is useful to improve the diagnostic sensitivity of endometrial neoplasms. However, caution must be taken when cancer-associated mutation(s) are detected in the genetic analysis. Further investigation may clarify the risk of malignant endometrial neoplasms in women with driver mutation(s).

## Background

Endometrial cancer (EC) is the most prevalent gynaecologic cancer, and the sixth and the eighth leading cause of cancer death among women in the United States and Europe, respectively [1]. Although the survival rate of localised EC is approximately 95.0%, the prognosis of metastatic EC remains unsatisfactory; the survival rate of EC with lymph node involvement is 69.8% and that with distant metastasis is as low as 18.4% according to the data of uterine cancer in Cancer Stat Fact from the National Cancer Institute [2]. These data indicate that the detection of EC at an early stage is crucial for successful treatment and/or cure of EC.

Risk factors of ECs include high body mass index (BMI) or obesity (BMI > 30), diabetes, hypertension, prolonged exposure to oestrogen (menarche at a young age, nulliparity and infertility, menopause at an older age, and polycystic ovarian syndrome), and use of tamoxifen [3, 4]. Women with Lynch syndrome have a high lifetime risk of EC, and those women are thus recommended to be enrolled in a surveillance programme.

Although mass screening has not been recommended for asymptomatic women, individuals with abnormal vaginal bleeding or discharge, and those with abnormal findings in transvaginal ultrasound imaging undergo endometrial screening. Endometrial cytology by brush sampling has been widely applied for the screening [5] because this method is a simple, painless, and inexpensive. LBC-based cytology represents an opportunity to overcome the obstacles in cytology and additionally enables to obtain high-quality DNA from the samples.

To increase the sensitivity of cytological screening of EC, we previously analysed 48 liquid cytology samples obtained for the cytological screening by both cytology and amplicon sequencing of five genes frequently mutated in EC. Although the genetic analysis improved the diagnostic sensitivity of EC screening [6], we found a pathogenic *KRAS* mutation in a healthy woman without any gynaecological neoplasms. To evaluate further the effectiveness of genetic analysis and determine the frequency of pathogenic mutations in women without malignant or premalignant diseases, we analysed in this study a total of 208 endometrial liquid cytology samples by Cancer Hotspot Panel containing 50 cancer-related genes. As a result, we corroborated that the combination of cytological and genetic analysis augmented the sensitivity of detection of malignant tumours. Intriguingly, we detected pathogenic alterations in 32 of 125 women without malignant or premalignant gynaecological neoplasms, which raises caution for the assessment of pathogenic mutation(s) in endometrial samples. It is an important clinical challenge to clarify whether the mutation-positive women without malignant or premalignant diseases may have high-risk for gynaecological malignancies.

## Materials and methods

### Selection of samples and clinical information

A total of 218 individuals were enrolled in this study, and liquid-based cytology (LBC) samples were collected by endometrial screening at Sapporo Medical University Hospital from October 2017 to March 2018. This study was approved by the institutional review boards of Sapporo Medical University (SMU-IRB, 292-77) and Institute of Medical Science, the University of Tokyo (IMSUT-IRB, 29-52-A1123). Written informed consent was obtained from all participants. All clinical investigation was conducted according to the principles expressed in the Declaration of Helsinki. We selected 208 samples from which we successfully extracted more than 200 ng of DNA, and these subjects were applied for genetic analysis. Among the 208 samples, 13 samples with low sequence coverage (<100-fold mean coverage) in the panel sequencing were excluded, and the remaining 195 were used for further analysis (Supplementary Table S1). The diagnosis of the 195 subjects is shown in Table 1. Endometrial tissues were obtained from two patients (subjects #39 and #168) who developed endometrial cancer during follow-up and underwent surgery.

**Table 1:** Cytology and mutation data of the 195 LBC samples.

Category of diagnosis	Histological classification (number of cases)	Cytology		Genetic analysis	
		Malignant or suspicious	Negative	Driver mutation (+)	Driver mutation (−)
Primary endometrial malignancy (*n* = 39)	Endometrial carcinoma				
	Endometrioid carcinoma (33)	20	13	25	8
	Carcinosarcoma (2)	2	0	2	0
	Clear cell carcinoma (2)	1	1	1	1
	Mucinous carcinoma (1)	1	0	1	0
	Endometrial sarcoma				
	Low-grade endometrial stromal sarcoma (1)	0	1	0	1
Non-endometrial malignancy (*n* = 15)	Cervical cancer (8)	6	2	5	3
	Ovarian cancer (6)	2	4	1	5
	Metastatic cancer (1)	1	0	1	0
Premalignant disease (*n* = 16)	Endometrial hyperplasia (9)	0	9	6	3
	Endometrial polyp(s) (7)	0	7	2	5
Benign/no disease (*n* = 125)	Myoma uteri/adenomyosis (66)	0	66	17	49
	CIN (11)	0	11	4	7
	Benign ovarian tumour (13)	0	13	3	10
	No disease (35)	0	35	8	27
Total (*n* = 195)		33	162	76	119

### Sampling and cytological examination

Endometrial LBC was performed by the standard procedure. Briefly, Screebrush (Soft Medical, Tokyo, Japan) was gently inserted to the level of the uterine fundus. The sample was placed into 10 ml of LBC PREP solution (Muto Pure Chemicals, Tokyo, Japan), and 8 ml of the liquid was used for cytological diagnosis and the remaining 2 ml was used for genetic analysis. Cytological diagnosis was carried out and evaluated according to the guideline provided by The Japanese Society of Clinical Cytology.

### DNA extraction and targeted sequencing

DNA was extracted from the 2 ml of LBC samples as described previously [6]. DNA was also extracted from FFPE samples of the two patients (subjects #39 and #168) using a QIAamp DNA FFPE Tissue Kit (Qiagen, Hilden, Germany). The sections of FFPE samples were prepared, and tumourous and non-tumourous components were separately collected by scalpel blade to enrich each component. Ten nanograms of DNA was applied for multiplex PCR amplification with AmpliSeq for Illumina Cancer HotSpot Panel v2 covering 207 areas in 50 oncogenes and tumour suppressor genes (Illumina, San Diego, CA). The amplicons were then digested, barcoded with AmpliSeq for Illumina CD Indexes Set A (Illumina), and purified using Agencourt AMPure XP (Beckman Coulter Inc). After the amplification and cleanup of the amplicons, sequencing was performed using an Illumina MiSeq platform according to the manufacturer’s protocol. Sequence data were aligned to human reference genome (hg19) and analysed using Local Run Manager. Variant Frequency Emit Cutoff >0.01 was used for variant call.

### Statistical analysis

Pearson’s chi-square test and Mann–Whitney *U-*test were used for statistical analyses with significance level considered at *P* < 0.05. All analyses were conducted using SPSS (IBM Corp, Armonk, NY).

## Results

### Diagnosis of the 195 subjects and cytological analysis

**Fig. 1:**
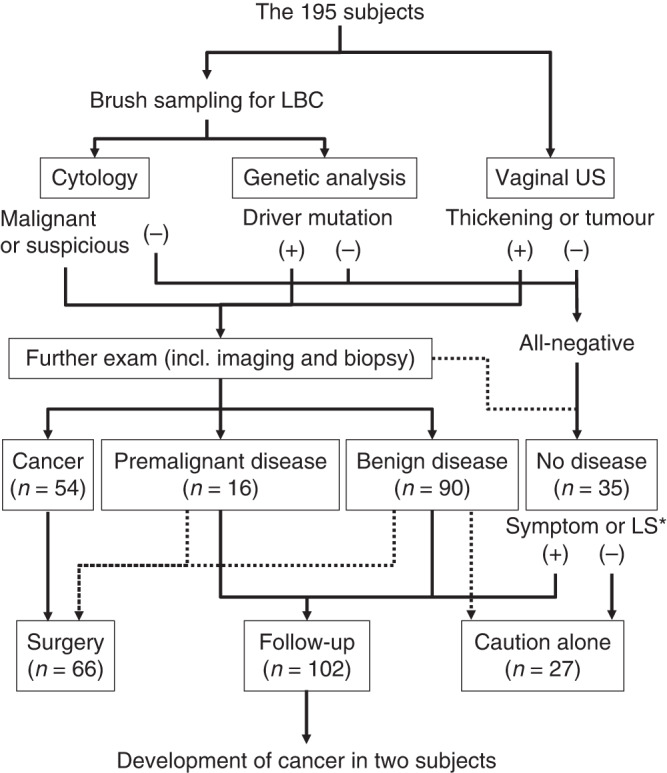
Flowchart of the screening, diagnosis, and post-diagnostic healthcare of the 195 subjects. *LS Lynch syndrome.

**Fig. 2:**
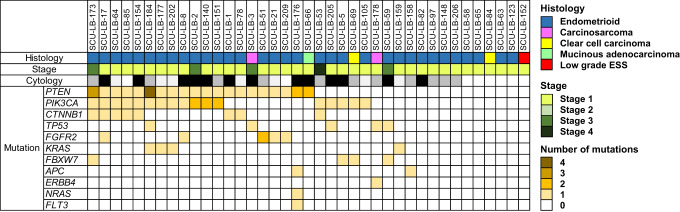
Cytological and genetic analyses of the 39 samples with primary endometrial malignancy. The colour of mutation column represents the number of pathogenic/likely-pathogenic mutations in the sample. Histological classification of the sample and the stage of disease are shown in the colour chart. Cytological diagnosis: positive in black, suspicious in grey, and negative in white.

Screening and diagnostic strategy of the 195 subjects is shown in Fig. 1. Thirty nine of the 195 were eventually shown to have primary endometrial neoplasms including 38 endometrial cancer and one endometrial sarcoma (Table 1). In addition, 15 were diagnosed to have non-endometrial malignancy (eight cervical cancer, six ovarian cancer, and one metastatic cancer), and 16 to harbour premalignant diseases (nine endometrial hyperplasia and seven polyps). Cytological investigation revealed that 33 of the 195 samples were positive (*n* = 19) or suspicious (*n* = 14) for malignancy. Twenty-four of the 33 were endometrial malignancy, and the remaining nine were non-endometrial cancer (Table 1 and Fig. 2). Collectively, 24 of 39 primary endometrial malignancies were positive or suspicious by cytology, indicating that the sensitivity was 61.5%. The 16 subjects with premalignant endometrial diseases and the 125 without malignant or premalignant diseases were all negative for cytology.

### Genetic analysis of the 195 LBC samples

Genetic analysis of the 195 samples identified at least one pathogenic mutation in 76 samples (Table 1). Pathogenic mutations were identified in 29 of the 39 women with primary endometrial malignancy, suggesting that the sensitivity was 74.4% (Fig. 2). In addition, mutations were found in seven of the 15 non-endometrial cancer (Table 1). Although genetic analysis alone overlooked 10 of the 39 endometrial malignancies, combination of cytology with genetic analysis diagnosed 33 of 39 primary endometrial malignancies, increasing the sensitivity to 84.6% (Fig. 2). The specificity of genetic analysis was 74.4% because mutations were not identified in 93 of the 125 women without malignant or premalignant gynaecological diseases.

The 29 mutation-positive primary endometrial tumours were all ECs and the number of mutations per case ranged from one to seven, and the average was 2.7 (Fig. 2 and Supplementary Table S2). The pathogenic mutations included mutations in *PTEN* (20 cases), *PIK3CA* (17 cases), *CTNNB1* (eight cases), *TP53* (five cases), *FGFR2* (five cases), *KRAS* (four cases), *FBXW7* (four cases), *APC* (two cases), *ERBB4* (one case), *NRAS* (one case), and *FLT3* (one case) (Supplementary Tables S2 and S3). The remaining ten mutation-negative endometrial malignancies included nine ECs (eight endometrioid cancer and one clear cell carcinoma) and one low-grade endometrial stromal sarcoma. In addition, we identified driver mutations in seven of 15 non-endometrial cancer (Table 1 and Supplementary Table S4).

Regarding the 16 cases with premalignant endometrial diseases, pathogenic mutations were identified in six of the nine endometrial hyperplasia, and two of the seven endometrial polyps (Supplementary Fig. 1 and Supplementary Table S5). The number of mutations per case in the eight mutation-positive premalignant lesions ranged from one to three, and the average was 2.3.

### Pathogenic mutations in the cases without malignant or premalignant gynaecological neoplasms

**Fig. 3:**
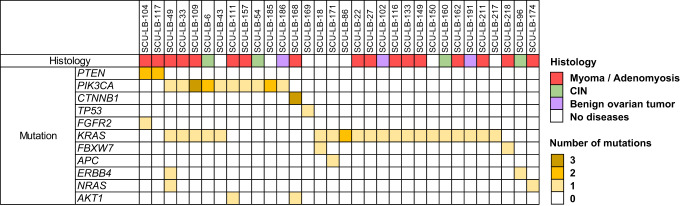
Pathogenic mutations in the 32 samples without malignant or premalignant diseases. The colour of mutation column represents the number of pathogenic/likely-pathogenic mutations in the sample. Histological classification of the sample is shown in the colour chart.

Importantly, we identified pathogenic variants in 32 of the 125 women without malignant or premalignant gynaecological diseases (Table 1). These mutations were found in *KRAS* (20 cases), *PIK3CA* (ten cases), *PTEN* (two cases), *AKT1* (two cases), *ERBB4* (two cases), *NRAS* (two cases), *TP53* (one case), *CTNNB1* (one case), *FGFR2* (one case), and *APC* (one case), suggesting that the identification of pathogenic variants in the LBC samples does not always predict the existence of malignant or premalignant lesion(s) in the endometrium (Fig. 3 and Supplementary Table S6). The number of variants per case in the 32 mutation-positive samples ranged from one to four, and the average was 1.5. It is of note that 13 of the 32 samples carried two or more mutations. Interestingly, we found three different *PIK3CA* mutations in a sample (subject #109), two different *PIK3CA* mutations in two samples (subjects #6 and #185), three *CTNNB1* mutations in a sample (subject #168). The three *CTNNB1* mutations (c.65_100del, c.94G > C, and c.100G > A) were in different sequence reads. Additionally, the sample from subject #86 had two different *KRAS* mutations at the same nucleotide position (c.35G > T and c.35G > A). These data indicated that endometrial samples should contain multiple cell clones. The median age of the cases with pathogenic variant was 56 years, and that without pathogenic variant was 54 years (Supplementary Table S7).

### Comparison of the frequencies of mutations among the mutation-positive samples

Naturally, frequency of the driver mutations found in women without malignant or premalignant diseases was lower than that in patients with malignant endometrial neoplasms. For example, *PIK3CA* mutations were found in ten (8.0%) of the 125 women without malignant or premalignant diseases, and in 17 (43.6%) of the 39 women with endometrial malignancy, suggesting that the frequency is approximately 5.4-fold higher in women with endometrial malignancy than those without (Table 2). However, *KRAS* mutations were found in 20 (16%) of the 125 women without malignant or premalignant diseases and in four (10.3%) of the 39 women with primary endometrial malignancies, implying that *KRAS* mutations should not be used for a biomarker of endometrial malignancies. In contrast, *PTEN* mutations were detected in two (1.6%) of the 125 women without malignant or premalignant diseases but were found in 20 (51.3%) of the 39 patients with endometrial malignancies, suggesting that the frequency is approximately 32-fold higher in women with endometrial malignancy than those without. Additionally, *CTNNB1* and *TP53* mutations were identified in single cases (0.8%) among the 125 individuals without malignant or premalignant diseases but were found in eight (20.5%) and five (12.8%), respectively, among the 39 women with endometrial malignancy, exhibiting that the frequencies are more than 16-fold higher in women with endometrial malignancy than those without malignancy. Therefore, *PTEN, CTNNB1* and *TP53* mutations may be useful for the biomarkers to predict endometrial malignancies.

**Table 2:** Frequency of mutations.

Gene	Patients with primary endometrial malignancies	Individuals without malignant or premalignant diseases
*PTEN*	20 (51.3%)	2 (1.6%)
*CTNNB1*	8 (20.5%)	1 (0.8%)
*TP53*	5 (12.8%)	1 (0.8%)
*PIK3CA*	17 (43.6%)	10 (8.0%)
*KRAS*	4 (10.3%)	20 (16%)

### Development of endometrial cancer in two women during follow-up

During the follow-up survey of 102 subjects without malignant neoplasms (Fig. 1), a 31-year-old woman (subject #39) developed stage 1A endometrial adenosarcoma 34 months after the diagnosis of endometrial polyps. In addition, a 51-year-old woman (subject #168) with myoma uteri without thickening of the endometrium at the first consultation suffered from abnormal uterine bleeding two years and nine months after the initial screening. Detailed examination found stage 1A endometrial cancer (endometrioid carcinoma grade1).

### Comparison of mutations between the initial LBC samples and cancer tissues

To investigate whether the endometrial cancer in subject #39 originated from the cell clone with the *CTNNB1* mutation (c.109T > G) detected in the initial LBC screening, we carried out genetic analysis of her cancer tissue. As a result, we identified a *CTNNB1* mutation (c.97T > G) that was different from the *CTNNB1* mutation (c.109T > G) in the LBC (Supplementary Table S8). Additional deep sequencing did not find the LBC mutation (c.109T > G) in the cancer tissue, or the cancer mutation (c.97T > G) in the LBC sample, indicating that the cancer cells were not originated from the cell clone captured by LBC.

In addition, genetic analysis of the cancer tissue in subject #168 identified two pathogenic *PTEN* mutations (c.389G > A and c.981del) and a *PIK3CA* mutation (c.3140A > G) (Supplementary Table S7). Again, none of the three *CTNNB1* (c.65_100del, c.94G > C, and c.100G > A) and *AKT1* (c.49G > A) mutations in the initial LBC sample were found in the tumour tissue by deep sequencing. Additionally, the *PTEN* or *PIK3CA* mutations were not detected in the LBC sample. These data suggested that the cancer cells did not develop from the clones with the *CTNNB1* or *AKT1* mutation(s).

Since the cancer tissue of subject #168 was endometrioid carcinoma (Supplementary Fig. 2A) accompanied by adjacent atypical hyperplasia (Supplementary Fig. 2B, C), we additionally analysed the hyperplasia and three regions with non-tumourous epithelium (Supplementary Fig. 2D–F). As a result, we identified the *PTEN* mutations (c.389G > A and c.981del) and *PIK3CA* mutation (c.3140A > G) in the atypical hyperplasia (regions #9HP and #11HP) but not in the regions with non-tumourous epithelium (regions #9N, #10N, and #12N), suggesting that the cancer developed from the cells in atypical hyperplasia. It is of note that three adjacent regions with non-tumourous epithelium carried different driver mutations; *PTEN* (c.57del) and *FBXW7* (c.1513C > T) in region #9N, *PIK3CA* (c.3132T > A) and *NOTCH1* (c.4732_4734del) mutations in region #10N, *FBXW7* (c.1310G > T) and *ATM* (c.8806G > T) mutations in region #12N (Supplementary Table S8). Taken together, the endometrium of subject #168 was covered with multiple cell clones carrying different driver mutations, and one of them probably transformed to endometrial cancer via atypical hyperplasia.

## Discussion

In this study, we extended our previous study and analysed a total of 195 LBC samples using a cancer hotspot panel containing 207 hotspot regions of 50 oncogenes or tumour suppressor genes. As a result, we corroborated that the genetic analysis increases the sensitivity of cytological screening for EC from 61.5% to 84.6%. For screening, the sensitivity is clinically more important than the specificity (74.4%). In addition, we identified driver alterations in eight of the 16 (50%) samples with premalignant neoplasms such as endometrial hyperplasia and polyps, all of which were not detected by cytology alone.

We also identified driver mutation(s) in 32 of 125 (25.6%) subjects without malignant or premalignant diseases. Several groups reported the detection of somatic mutations in normal endometrium and precancerous lesions. Wang and colleagues identified mutations in only nine pap brush samples from 714 women without cancer [7]. In stark contrast of this report, Maritschnegg et al. identified *KRAS* mutations in six (22.2%) of 27 women with benign lesions by genetic analysis of uterine lavage samples [8]. They extended the study and identified somatic mutations in 51 (53.7%) of 95 lavage samples from women without malignant endometrial diseases [9]. They also revealed that increasing age and post-menopausal status were associated with the presence of these cancer-associated mutations.

Recent studies disclosed that the non-tumourous endometrial glandular epithelium harbored multiple clonal cell populations carrying driver mutations in cancer associated genes. Suda et al. analysed 107 ovarian endometriotic samples and 82 histologically normal uterine endometrial epithelium samples by panel sequencing and identified somatic mutations in 71 of the 82 normal samples [10]. They further sequenced 109 single endometrial glands and found that each gland carried distinct cancer-associated mutations, demonstrating the heterogeneity of the genomic architecture of endometrial epithelium. Moore et al. analysed a total of 292 histologically normal endometrial glands from 28 women by whole genome sequencing coupled with laser microdissection [11]. As a result, they found somatic mutations ranged from 209 to 2,833 substitutions in the 292 glands, and showed that 147 out of 257 endometrial glands carried at least one driver mutation. Taken together, endometrial epithelium is composed of multiple glands with clonal cell populations accumulating distinct somatic mutations. The identification of multiple driver mutations in the oncogenes including *PIK3CA*, *CTNNB1*, and *KRAS* in the women without malignant or premalignant neoplasms is in complete agreement with these reports.

In addition, previous studies revealed that *PTEN*, *CTNNB1*, and *TP53* mutations were less frequent compared with *PIK3CA* mutations; Suda et al. identified *PTEN* (three cases), *CTNNB1* (one case), *TP53* (one case) and *PIK3CA* (12 cases) driver mutations in 29 healthy women [11], and Moore et al. identified *PTEN* (two cases), *CTNNB1* (no case), *TP53* (three cases) and *PIK3CA* (15 cases) driver mutations in 28 women without malignant diseases [10]. These data are consistent with our finding that frequencies of *PTEN*, *CTNNB1*, and *TP53* mutations were much less than those of *PIK3CA* mutations in women without malignant or premalignant diseases (Table 2), and support the notion that mutations in the *PTEN*, *CTNNB1*, and *TP53* genes may be useful for the biomarkers of high risk of developing malignant endometrial tumours. This notion is exemplified by the development of endometrial cancer in two woman (subject #39 and #168) with *CTNNB1* mutation(s) in our follow-up survey.

To our surprise, we found completely different mutations between the endometrial cancer tissues and the initial LBC samples in the two women. Since we could not collect all endometrial epithelial clones by brush sampling, we may have missed the mutations in the clones that later developed to the malignant neoplasms. Alternatively, the clones might have been lurked in the soil of endometrial epithelial forest as a dormant state. Since the driver mutations in the tumour of subject#168 included *PIK3CA* c.3140A > G (VAF: 0.169), *PTEN* c.389G > A (VAF: 0.166) and *PTEN* c.981del (VAF: 0.125), the second hit of the *PTEN* deletion might have transformed the non-neoplastic clone to the premalignant and/or malignant clones. Since the cancer was surrounded by non-tumourous epithelium clones carrying driver mutations such as *PTEN* (c.57del) and *FBXW7* (c.1513C > T), *PIK3CA* (c.3132T > A) and *NOTCH1* (c.4732_4734del), *FBXW7* (c.1310G > T) and *ATM* (c.8806G > T), these combinations or genetic status alone might not be enough to transform the clones to premalignant or malignant neoplasms. Regarding the subject #39, the initial screening detected a clone containing the mutation in *CTNNB1* (c.109T > G), but the cancer developed from a clone carrying a different *CTNNB1* mutation (c.97T > G); both mutations are located at the GSK3β phosphorylation sites and are thus supposed to render resistance to its degradation. This case raises another important issue as to what causes the difference between normal cells with a driver mutation(s) and tumour cells with an equivalent mutation(s). Although other genetic factors such as structural variants, copy number change and mutations in other cancer-associated genes may be involved in the transformation of endometrial clones, we may need to consider non-genetic factors such as epigenetic and environmental factors.

It is of note that the epithelial stem/progenitor cells residing in the basalis layer of the endometrium, differentiate and proliferate up to the exposed surface in response to elevated oestrogen levels [12], where they are shed into the cavity of the uterus. Thus, if a driver mutation is acquired in the stem/progenitor cells, its offspring clone(s) carrying the mutation will be retained in the epithelial layer and proliferate in the endometrial glands even after menstruation. However, if a driver mutation is acquired in the differentiated cells, the cell clone may be shed out from the epithelial layer by menstruation and not stay in the remodelled endometrial glands. Thus, repeated genetic screening may help to identify genetic changes carried in epithelial stem cells. It is noteworthy that the average number of mutations gradually increased from subjects without malignant or malignant diseases to those with malignant neoplasm (1.5 mutations in cases without malignant or malignant diseases, 2.3 with premalignant diseases, and 2.7 with malignant neoplasm). Therefore, existence of multiple non-cancerous clones with accumulated driver mutations may be a high risk for the malignant transformation as shown in the non-malignant clones in subject #168.

The greatest advantage of genetic analysis is the increase of sensitivity in the endometrial screening. Since cytological and genetic analysis can be done using the same LBC sample, there may be no need for the participants to undergo additional invasive examination(s). Although the cost of genetic analysis is a disadvantage, the total cost of treatment for advanced or recurrent diseases may be reduced by early detection of malignant and premalignant endometrial diseases. In addition, the cost of genetic analysis will decrease as cancer profiling tests become more popular. Furthermore, inclusion of responsible genes for familial cancer such as mismatch repair genes and *BRCA1/2* in the amplicon sequencing may be useful for the identification of patients with Lynch syndrome or HBOC, because the genetic analysis detects not only somatic mutations but also germ line variants. Although the specificity of genetic analysis of endometrial brush samples was reported as high as 99% in a previous report [7], the specificity was 74.4% in this study. This decreased specificity may result from the detection of minor non-malignant cell clones carrying cancer-associated mutation(s) because the average mean coverage of our amplicon sequencing was as high as 1181 (Supplementary Table S1). Therefore, we should be careful not to over-diagnose mutation-positive cases as malignant diseases since the specificity of genetic analysis may not be close to 100%. Hence, it is a challenge how to assess and deal with mutation-positive subjects who do not show any abnormalities by other analyses such as cytology and transvaginal ultrasound imaging.

In conclusion, we have disclosed in this study that mutations in *PTEN*, *CTNNB1*, *TP53*, or multiple genetic alterations may be associated with high-risk condition for the development of malignant endometrial neoplasms. In addition, repeated genetic analysis may be useful to identify genetic changes in the epithelial stem/progenitor cells. Although caution should be warranted when cancer-associated somatic mutation(s) are detected in the genetic analysis, careful investigation and follow-up survey may reduce the mortality of EC by the early detection of malignant neoplasms.

## Supplementary information

Supplementary Figure 1

Supplementary Figure 2

Supplementary Tables

## Data Availability

The datasets generated and/or analysed during the current study are available from the corresponding author on reasonable request.

## References

[1] Urick ME, Bell DW. Clinical actionability of molecular targets in endometrial cancer. Nat Rev Cancer. 2019;19:510–21. 31388127 10.1038/s41568-019-0177-x PMC7446243

[2] Surveillance, Epidemiology, and End Results Program 22 (2013-2019) at https://seer.cancer.gov/statfacts/html/corp.html.

[3] Oaknin A, Bosse TJ, Creutzberg CL, Giornelli G, Harter P, Joly F, et al. Endometrial cancer: ESMO clinical practice guideline for diagnosis, treatment and follow-up. Ann Oncol. 2022;9:860–77. 10.1016/j.annonc.2022.05.009 35690222

[4] Colombo N, Creutzberg C, Amant F, Bosse T, González-Martín A, Ledermann J, et al. ESMO-ESGO-ESTRO Consensus Conference on Endometrial Cancer: diagnosis, treatment and follow-up. Ann Oncol. 2016;1:16–41. 10.1093/annonc/mdv484 26634381

[5] Fulciniti F, Yanoh K, Karakitsos P, Watanabe J, Lorito AD, Margariet N, et al. The Yokohama system for reporting directly sampled endometrial cytology: the quest to develop a standardized terminology. Diagn Cytopathol. 2018;46:400–12. 29479846 10.1002/dc.23916

[6] Matsuura M, Yamaguchi K, Tamate M, Satohisa S, Teramoto M, Iwasaki M, et al. Efficacy of liquid-based genetic diagnosis of endometrial cancer. Cancer Sci. 2018;109:4025–32. 30289582 10.1111/cas.13819 PMC6272085

[7] Wang Y, Li L, Douville C, Cohen JD, Yen TT, Kinde I, et al. Evaluation of liquid from the Papanicolaou test and other liquid biopsies for the detection of endometrial and ovarian cancers. Sci Transl Med. 2018;433:eaap8793. 10.1126/scitranslmed.aap8793 PMC6320220 29563323

[8] Maritschnegg E, Wang Y, Pecha N, Horvat R, Nieuwenhuysen EV, Vergote I, et al. Lavage of the uterine cavity for molecular detection of mullerian duct carcinomas: a proof-of-concept study. J Clin Oncol. 2015;33:4293–4300. 26552420 10.1200/JCO.2015.61.3083 PMC4678180

[9] Nair N, Camacho-Vanegas O, Rykunov D, Dashkoff M, Camacho SC, Schumacher CA, et al. Genomic analysis of uterine lavage fluid detects early endometrial cancers and reveals a prevalent landscape of driver mutations in women without histopathologic evidence of cancer: a prospective cross-sectional study. PLoS Med. 2016;13:e1002206. 28027320 10.1371/journal.pmed.1002206 PMC5189938

[10] Suda K, Nakaoka H, Yoshihara K, Ishiguro T, Tamura R, Mori Y, et al. Clonal expansion and diversification of cancer-associated mutations in endometriosis and normal endometrium. Cell Rep. 2018;24:1777–89. 30110635 10.1016/j.celrep.2018.07.037

[11] Moore L, Leongamornlert D, Coorens THH, Sanders MA, Ellis P, Dentro SC, et al. The mutational landscape of normal human endometrial epithelium. Nature. 2020;580:640–6. 32350471 10.1038/s41586-020-2214-z

[12] Cousins FL, Pandoy R, Jin S, Gargett CE. The elusive endometrial epithelial stem/progenitor cells. Front Cell Dev Biol. 2021;9:640319. 33898428 10.3389/fcell.2021.640319 PMC8063057

