# Supplementary material for: Identification of cancer driver mutations in liquid-based cytology samples for the screening of endometrial diseases
Source: BJC Rep. 2023 Nov 2;1:18. doi: 10.1038/s44276-023-00020-y (PMC11524081; doi:10.1038/s44276-023-00020-y)
Supplement: Supplementary file 1 — Supplementary Figure 1 [file 44276_2023_20_MOESM1_ESM.pptx]

## Slide 1
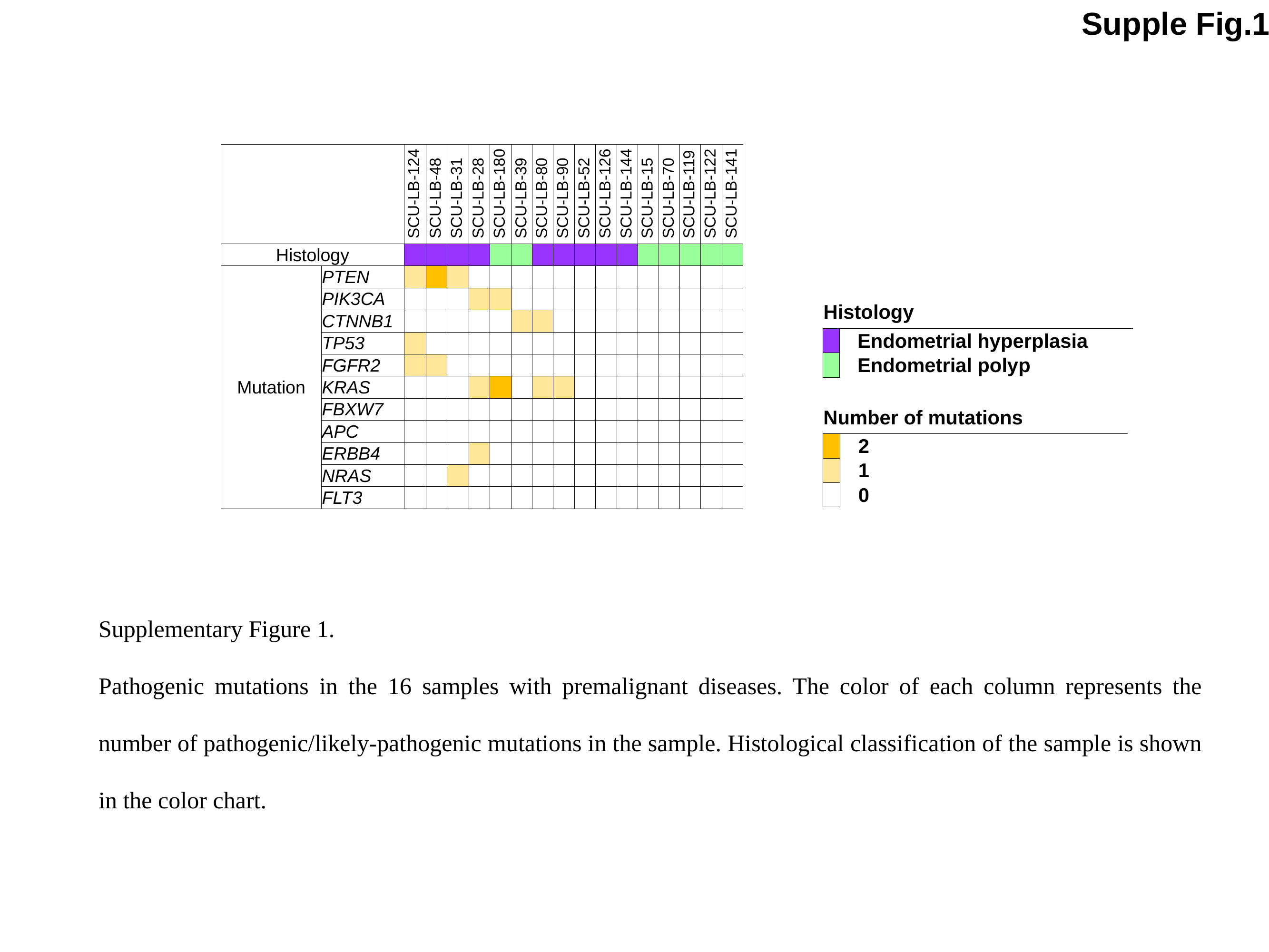

Supple Fig.1
| | | SCU-LB-124 | SCU-LB-48 | SCU-LB-31 | SCU-LB-28 | SCU-LB-180 | SCU-LB-39 | SCU-LB-80 | SCU-LB-90 | SCU-LB-52 | SCU-LB-126 | SCU-LB-144 | SCU-LB-15 | SCU-LB-70 | SCU-LB-119 | SCU-LB-122 | SCU-LB-141 |
| --- | --- | --- | --- | --- | --- | --- | --- | --- | --- | --- | --- | --- | --- | --- | --- | --- | --- |
| Histology | | | | | | | | | | | | | | | | | |
| Mutation | PTEN | | | | | | | | | | | | | | | | |
| | PIK3CA | | | | | | | | | | | | | | | | |
| | CTNNB1 | | | | | | | | | | | | | | | | |
| | TP53 | | | | | | | | | | | | | | | | |
| | FGFR2 | | | | | | | | | | | | | | | | |
| | KRAS | | | | | | | | | | | | | | | | |
| | FBXW7 | | | | | | | | | | | | | | | | |
| | APC | | | | | | | | | | | | | | | | |
| | ERBB4 | | | | | | | | | | | | | | | | |
| | NRAS | | | | | | | | | | | | | | | | |
| | FLT3 | | | | | | | | | | | | | | | | |
| Histology | | | | | | | | | |
| --- | --- | --- | --- | --- | --- | --- | --- | --- | --- |
| | | Endometrial hyperplasia | | | | | | | |
| | | Endometrial polyp | | | | | | | |
| Number of mutations | | | |
| --- | --- | --- | --- |
| | 2 | | |
| | 1 | | |
| | 0 | | |
Supplementary Figure 1.
Pathogenic mutations in the 16 samples with premalignant diseases. The color of each column represents the number of pathogenic/likely-pathogenic mutations in the sample. Histological classification of the sample is shown in the color chart.
