# Supplementary material for: Identification of cancer driver mutations in liquid-based cytology samples for the screening of endometrial diseases
Source: BJC Rep. 2023 Nov 2;1:18. doi: 10.1038/s44276-023-00020-y (PMC11524081; doi:10.1038/s44276-023-00020-y)
Supplement: Supplementary file 2 — Supplementary Figure 2 [file 44276_2023_20_MOESM2_ESM.pptx]

## Slide 1
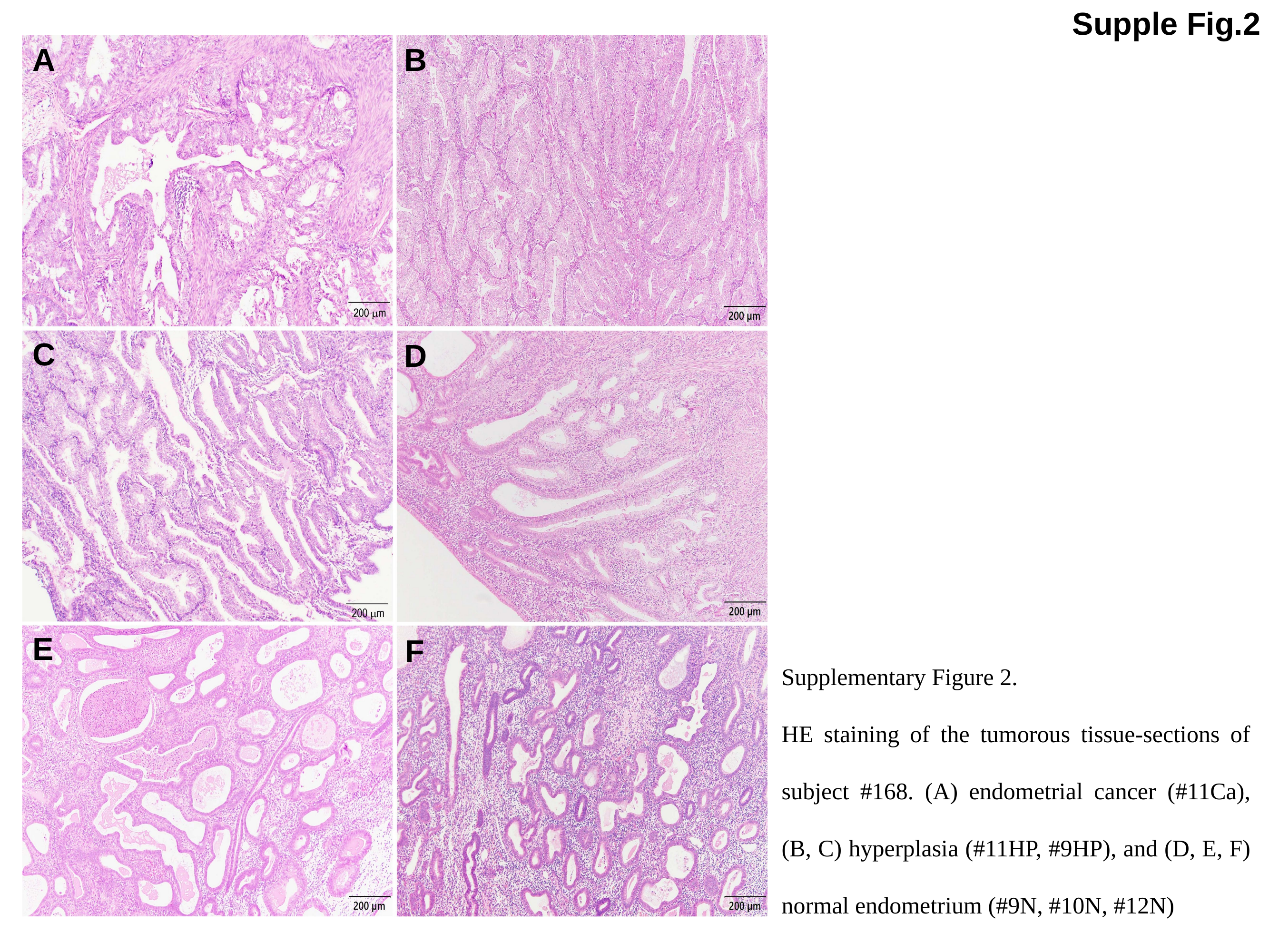

Supple Fig.2
A
B
C
D
E
F
Supplementary Figure 2.
HE staining of the tumorous tissue-sections of subject #168. (A) endometrial cancer (#11Ca), (B, C) hyperplasia (#11HP, #9HP), and (D, E, F) normal endometrium (#9N, #10N, #12N)
